# Insights from Turkey's big data: unraveling the preventability, pathogenesis, and risk management of Alzheimer's disease (AD)

**DOI:** 10.1038/s41598-024-56702-1

**Published:** 2024-03-12

**Authors:** Talip Yiğit, Naim Ata, Murat Dinçer, M. Mahir Ülgü, Şuayip Birinci, M. Okan Ayvalı

**Affiliations:** 1 Faculty of Economics and Administrative Sciences, Istanbul 29 Mayıs University, Ümraniye, İstanbul, Turkey; 2 General Directorate of Health Information System, Turkish Ministry of Health, Ankara, Turkey; 3 Turkish Ministry of Health, Ankara, Turkey

**Keywords:** Alzheimer, Dementia, Chronic diseases, Connectivity, Alzheimer's disease, Dementia

## Abstract

Extensive research into dementia has more recently honed in on several key areas. These areas include the advancement of techniques such as the accumulation of amyloid-β and tau proteins, the monitoring of cerebral hypometabolism rates etc. The primary objective of this study is to explore the intricate interplay between Alzheimer's disease (AD)—other dementias (D) and various chronic illnesses in terms of time, intensity, and connectivity. In this context, we retrospectively examined data of 149,786 individuals aged 65 and above who received diagnoses of AD and D in the year 2020. At first, logistic regression (LR) analysis has been made with “sex”, “age” and “foreigner” (citizenship status) independent variables for AD and D. The LR models shows that while “sex” and “age” variables have a small rate on the risk of developing AD/D, it is detected that being a foreigner increase the risk of AD and D as 69.8% and 88.5% respectively. Besides, the LR models have middle-level success prediction rate for both of the two dependent variables. Additionally, we used the parallel coordinates graphs method within the R Studio to visualize their relationships and connections. The findings of this investigation strongly suggest that AD/D don’t stand as isolated conditions, but rather stem from intricate interactions and progressive processes involving diverse chronic diseases over time. Notably, ailments including hypertension, coronary artery disease, diabetes, hyperlipidemia, and psychological disorders, contribute substantially to the emergence of both AD and D. This study highlights that the fight against AD/D can only be possible with next-generation prophylactic interventions that can predict and manage risks. Such an approach holds the potential to potentially lower AD and dementia to levels that are amenable to treatment.

## Introduction

Dementia is a term that encompasses the adverse effects on memory, thinking, and behavior resulting from brain damage. Currently, it is estimated that around 55 million people are affected by at least one form of dementia. Of these individuals, nearly 60% reside in low- and middle-income countries^1^.

One of the most widely recognized forms of dementia is Alzheimer's disease (AD), which was first identified in 1906^2^. AD constitutes approximately 70% of all dementia cases and follows a progressive course, resulting in the gradual erosion of essential abilities across its early, middle, and late stages^3,4^. Various diagnostic methods including magnetic resonance imaging (MRI), positron emission tomography (PET) and analysis of cerebrospinal fluid are employed to diagnose AD. Notably, saliva and blood tests also stand among these diagnostic approaches^5,6^. Despite these extensive endeavors, a definitive cure for AD remains elusive^7^. The collective global expenditure for the development of diagnostic and therapeutic strategies for AD amounts to 1.3 trillion dollars annually^1^. Predictions indicate that this expense will surpass 9 trillion dollars by the year 2050^8^.

In addition to treatment endeavors, the significance of preventive measures against AD and dementia is underscored. Debates indeed suggest that up to 40% of all dementia cases could potentially be preventable or delayed^9^. The discussions on prevention and delay necessitate the determination of risk levels for individuals to develop demetia diseases in the pre-symptomatic stage. This has spurred a heightened interest in dementia biomarkers, especially within the context of AD. Notably, the accumulation of amyloid-β (Aβ), tau protein pathologies, cerebral hypometabolism, and structural brain alterations stand as significant biomarkers for AD and other dementia types. In a study conducted with a total of 200 participants, it has been shown that the accumulation of Aβ can be traced back up to 20 years before the diagnosis of dementia. Concurrently, evidence indicates that cerebral hypometabolism becomes apparent within a 10–15 year timeframe before diagnosis, while structural brain changes manifest within the final 5 years preceding diagnosis^10–12^. Similarly, indicators such as Tau-181 and T-Tau exhibit notably higher values in individuals diagnosed with AD compared to those unaffected by AD^5^. Furthermore, while tau values may exhibit certain intervals of increase during the post-diagnostic phase, no significant alteration is observed in Aβ accumulation^13^.

Additionally, the reactivation of astrocytes, which are essential components of normal brain function, is considered to be associated with Aβ accumulation and tau protein pathologies. It has also been revealed that astrocytes are a component of amyloid plaques. Based on this foundation, there is a discussion regarding the evaluation of astrocytes as another biomarker^14–16^.

The precursor factors for dementia, particularly Alzheimer's, are not limited to these chemical markers alone. Currently, the prevalence of various diseases in individuals diagnosed with dementia suggests a relationship between these diseases and dementia, specifically Alzheimer's. One such disease is hypertension. It is known that high blood pressure can damage the capillaries in the brain, leading to impairment in memory and cognitive functions.

Various studies have determined that high blood pressure is a risk factor for Alzheimer's and other forms of dementia, supporting this statement^17,18^. Furthermore, analysis of the results from 209 different studies revealed a positive relationship between hypertension and mental illnesses, including dementia^19^. In another study, it was found that at least 30% of individuals with hypertension had mild cognitive impairment (MCI)^20^. Similarly, a study involving 92 participants showed that individuals diagnosed with hypertension had significantly lower cognitive abilities^21^. Additionally, a meta-analysis examining studies conducted with over one and a half million individuals found that the use of antihypertensive medication was identified as a protective factor against Alzheimer's disease^22^.

Hyperlipidemia is also among the diseases associated with Alzheimer's. In a study conducted on 113 Alzheimer's patients in Japan, the prevalence of hypertension was found to be 42%, while lipid abnormalities were at a level of 48%^23^. In a study examining the impact of statin use on the likelihood of developing Alzheimer's, it was determined that statin use did not increase the risk of Alzheimer's diagnosis^24^. Another study found that an increase in high-density lipoprotein cholesterol (HDL) and hypertension resulting from genetic predisposition were associated with an increased risk of Alzheimer's^25^.

Diabetes, characterized by structural degeneration contrary to the functioning principle of insulin receptors highly concentrated in the hippocampus, is closely related to dementia^26^. Indeed, diabetes is commonly observed especially in individuals diagnosed with Alzheimer's and vascular dementia^27–29^. A study conducted with data from 466,000 individuals calculated that the risk of dementia was 190% higher in individuals diagnosed with diabetes before the age of 45^30^. These findings have been observed to support that having a diagnosis of diabetes in various studies increases the risk of dementia by 2–4 times^26^. Furthermore, it is known that high HbA1c levels can be associated with cognitive decline^31^. In a study that supports all of these findings, a total of 41 studies examining the relationship between dementia and diabetes, hypertension, cardiovascular diseases, depression, and gastrointestinal diseases have demonstrated a positive association between dementia and these conditions^32^.

In addition, various studies have shown that a history of depression increases the risk of dementia, and there is a positive relationship between the severity of depression and the likelihood of developing dementia^33,34^. Indeed, a depression diagnosis is known to be associated with receiving a dementia diagnosis within a span of up to 20 years^35^. Individuals diagnosed with depression have been observed to experience shrinkage in the hippocampus region of the brain, which serves as a significant reference in explaining the relationship between depression and Alzheimer's^36,37^. Another study found that individuals with a genetic predisposition to Alzheimer's are at a higher risk of experiencing depression during middle age^38^. Furthermore, the common relationship between oxidative stress, which leads to damage in the brain and the hyper-phosphorylation of amyloid-β and tau proteins, in depression and Alzheimer's suggests a potential link between these two conditions. Additionally, studies have shown that loneliness and social isolation increase the risk of dementia^34^.

Based on findings related to biomarkers, it becomes evident that the pre-diagnostic phase of AD begins to develop and when evaluating the relationship of various diseases with Alzheimer's, it becomes necessary to better understand the developmental nature of the pre-diagnostic process. Therefore, the purpose of this study is to examine the relationship between AD (ICD10 Codes of illnesses that included as AD are; F00, F00.0, F00.1, F00.2, F00.9, and G30.) and other forms of dementia (ICD10 Codes of illnesses that included as Dementia are; F01, F02, F02.0, F02.1, F02.2, F02.3, F02.4, F02.8, F03, and G31.) with various diseases within a specific time frame.

## Method

This study was conducted with the permission of the Ministry of Health of the Republic of Turkey. The data used in the study was extracted through the “e-nabız” system developed by the Ministry^39^. Data of individuals aged 65 and above who underwent AD/dementia screening in 2020 were retrospectively analyzed, and observations regarding the variables in the dataset related to these individuals were collected. All methods were carried out in accordance with relevant guidelines and regulations. The data set used in the analysis was obtained in accordance with the principles of the Personal Data Protection Law No. 6698 dated 24.3.2016 of the Republic of Turkey and archived by the research team. In addition, in obtaining the data set and carrying out the analysis, the principles of the Ethics Committee for Public Servants of the Ethics Commission of the Ministry were adhered to. The study was reviewed by commission officers and the compliance of the data set acquisition, analysis and reporting processes with scientific ethical rules was approved.

In the literature, there are studies that examine whether individuals in a specific sample experience various diseases and AD simultaneously. In contrast to those studies, our research aims to explore the relationships between individuals diagnosed with AD/dementia and the diseases they were diagnosed with before and after the AD/dementia diagnosis. All individuals in the dataset have received an AD/dementia diagnosis in the year 2020.

In this context, the first step is to examine the frequency of 11 different diseases in the dataset before (pre) and after (post) AD/dementia diagnosis. Subsequently, changes in the total number of pre and post diseases will be presented. Table 1 provides information related to these diseases.

**Table 1:** Investigated ıllnesses.

Illness	ICD 10	Abbreviation
Renal failure	N14 N19	renal.f
Heart failure	I50	heart.f
Atrial fibrillation	I48	af
Cerebrovascular disease	I64	cerebro
Coronary (artery)	I25.1	coronary
Diabetes mellitus	E10 E14	diabetes
Hyperlipidaemia	E78.2 E78.4 E78.5	h.lipid
Hypertension	I10 I15	h.tension
Chronic obstructive pulmonary disease	J44.9	copd
Osteoporosis	M80 M81 M82	osteoporosis
Psychological	F31 F32 F33 F34.1 F06.3	psy

In this study, logistic regression (LR) analysis was employed to investigate the impact of age, biological sex, and citizenship status on AD/dementia. For both datasets, individuals without a diagnosis of AD/dementia were randomly selected from the 'e-nabız' system. These individuals had previously undergone an AD/dementia inquiry and remained undiagnosed. Consequently, there were 87,591 undiagnosed patients in the AD dataset and 62,193 in the dementia dataset, with an equal number of diagnosed patients. The analysis was conducted on the data of 175.182 persons for ad and 124.386 persons for dementia in total. Prior to LR analysis,an outlier analysis was conducted for the continuous “age” variable which led to to the exclusion of 135 observations in the Alzheimer's dataset and 92 in the dementia dataset. Annex 1 provides detailed information on the data used for LR analysis. To enhance prediction validity, a test-train method was employed for LR analysis, splitting the data into an 80% training set and a 20% testing set. The 80–20% model yielded the highest prediction success for the Alzheimer's dataset, while the 70–30% model performed best for the dementia dataset.

In addition to LR analysis, the study utilized the Parallel Coordinates Graph (PCG) method. The main focus of the study is to examine the relationships between pre and post diseases, as well as their relationship with AD/dementia, using visual outputs obtained through the PCG. PCG outputs were obtained using the "plotly" package in R Studio 4.3.0^40^. Other graphical representations were created using Microsoft Power BI program.

In the figures presented in our research, the time period are limited for a proper visualisation. This limit determined as 10 years since there is a significant deposition of illnesses in the 10 years period. Therefore, observations related to IDs that developed the disease more than 120 months pre/post have been removed from the dataset. As a result, there was a data loss of 1.4% from the 'pre' dataset and 0.23% from the 'post' dataset. Consequently, the number of observations used in pre-disease PCG graphs is 87,065 for AD and 61,265 for dementia. For post-disease PCG graphs, the number of observations is 87,592 for AD and 62,194 for dementia. In total, the sum of observation number is 148.330 for “pre” process and 149.786 for “post” process.

The analyses within the scope of the study were conducted based on the time difference between the diagnosis dates of the diseases mentioned in Table 1 and the date individuals underwent Alzheimer's/dementia screening. The relevant time difference was calculated on a monthly basis. Diseases diagnosed with Alzheimer's/dementia screening up to 29 days before are encoded as "1" (diagnosed one month before) instead of the value "0," which indicates that they did not receive the corresponding pre-disease diagnosis.

Furthermore, these investigations will be further analyzed based on whether the individuals' geographical regions have AD/dementia rates above or below the national average for the 65 + age population. Accordingly, for individuals diagnosed with AD, those residing in the Mediterranean, Marmara, and Black Sea regions (a total of 53,436 individuals pre and 53,614 individuals post); and for individuals diagnosed with dementia, those living in the Aegean, Marmara, and Black Sea regions (a total of 39,992 individuals pre and 40,877 individuals post) have AD/dementia rates higher than the country's average in relation to the 65 + age population. The average of other regions is below the national average.

In addition, each line represents an individual, and the numerical values in the visuals are given in "months." The name of the respective variable is written at the top of each column, and the value below it represents the maximum value for that variable. The "0" values in the visuals reflect the absence of a diagnosis.

## Analysis

As part of the study, the prevalence of the relevant diseases in individuals diagnosed with AD and dementia has been examined proportionally. In this context, the rates of diseases that occurred before (pre) and after (post) AD/dementia diagnosis have been presented separately.

The most common disease among these individuals is hypertension. Additionally, except for the difference between renal failure and cerebrovascular disease, the prevalence of all other diseases is in the same order for both groups. It is noteworthy that diabetes, hyperlipidemia, hypertension, depression, and bipolar disorder are among the top five most common diseases.

**Figure 1:**
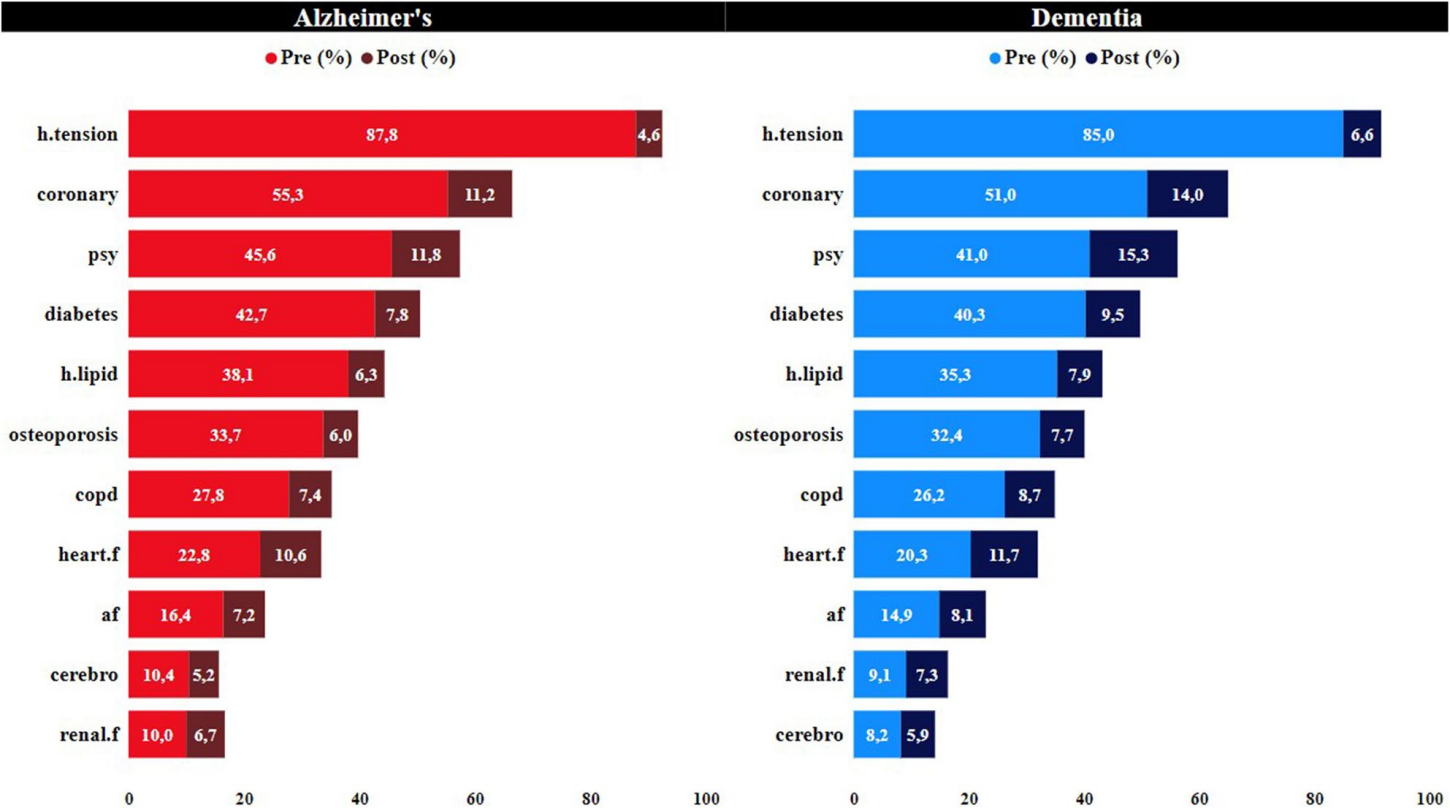
Prevalence of various diseases among individuals diagnosed with ad and dementia. The light-colored sections in the figure represent the prevalence of pre-diseases, while the dark-colored sections represent the prevalence of post-diseases. The values given in percentage (%) reflect the proportion of individuals in the respective group who experienced that disease. Hypertension is the most common disease with a significant difference in prevalence.

**Figure 2:**
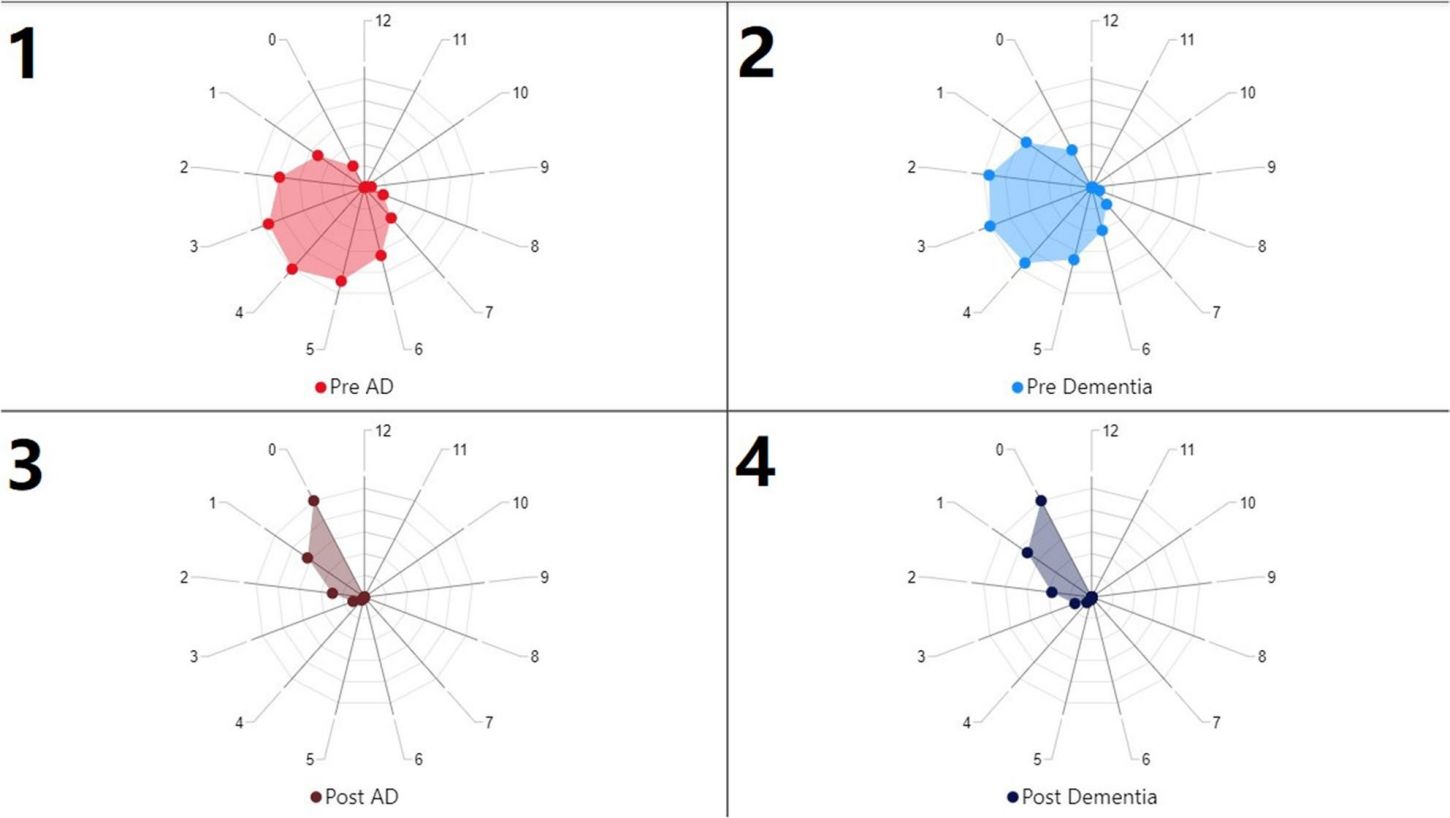
Prevalence of total pre and post diseases among individuals diagnosed with ad and dementia. Figure 2 consists of four different graphical representations. Graphs "1" and "3" present the distribution of the total number of pre and post diseases in individuals diagnosed with AD. According to this, individuals diagnosed with AD have a prevalence of 64% for having 2–5 pre-diseases, while 47% of them have no post-diseases, and only 30% have 1 post-disease. Information related to individuals diagnosed with dementia is presented in representations "2" and "4." From these representations, it can be observed that individuals diagnosed with dementia have a prevalence of 66% for having 2–5 pre-diseases. The prevalence of post-disease among individuals with dementia is 42% for having none and 30% for having 1 post-disease. As a summary of these four graphical representations, the prevalence of experiencing different diseases before the AD/dementia condition is high.

Figure 2 represents the distribution of total pre and post diseases separately for individuals in the two datasets. As can be understood from Fig. 2, the total number of pre-diseases is concentrated in the range of 2–5 for both groups. Thus, the likelihood of an individual diagnosed with AD/dementia having 2–5 pre-diseases before the diagnosis is high. This finding is consistent with the information obtained from the literature review and the results presented in Fig. 1. Another conclusion that can be drawn from Fig. 2 is related to the total number of post-diseases. For both groups, individuals who did not receive any disease diagnosis after the AD/dementia diagnosis have the highest proportion. Those who received 1 diagnosis after AD/dementia come in the second place, while the number of individuals with a total of 2 or more post-diseases is significantly lower.

**Figure 3:**
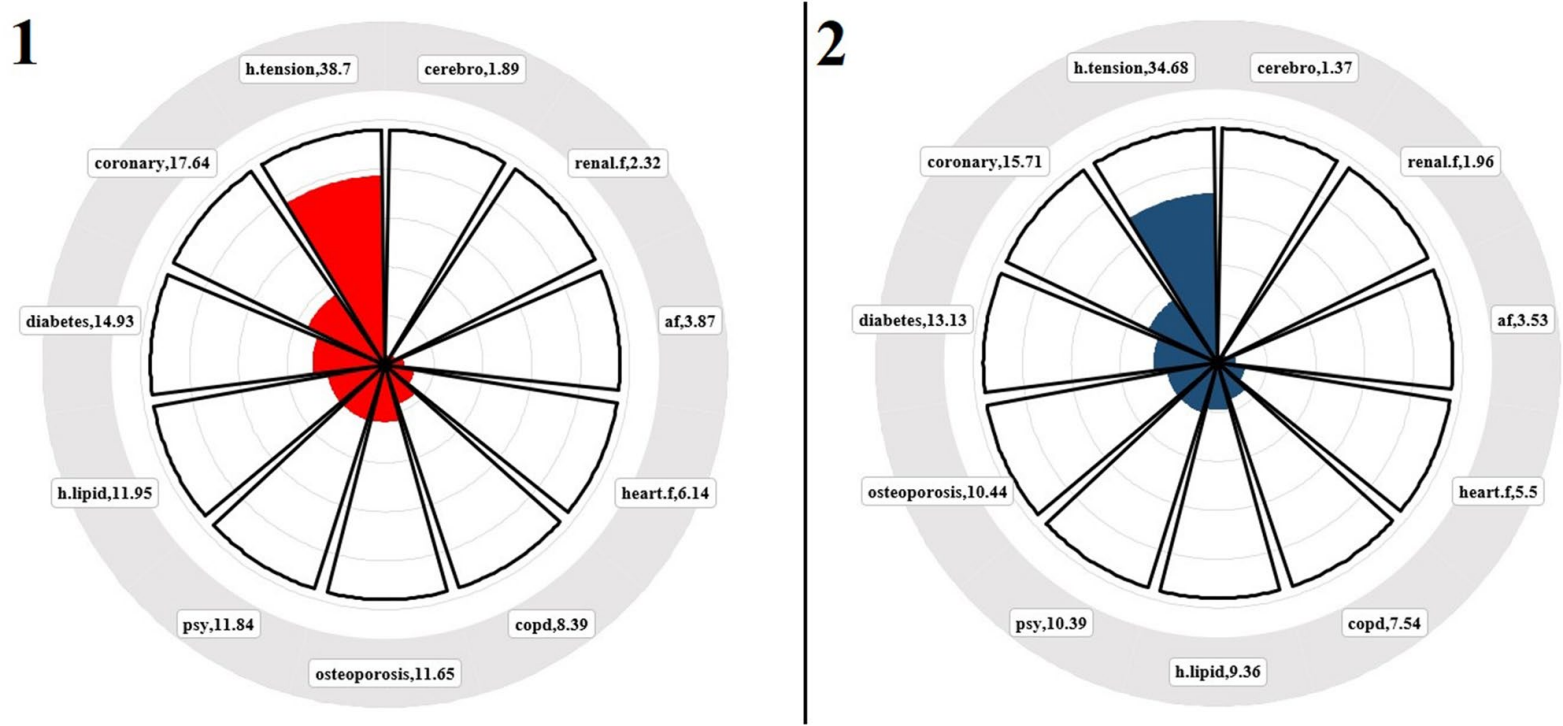
Time of onset of pre-diseases before AD/dementia diagnosis (Months). The visual labeled as "1" represents the distribution of pre-diseases just before AD, while the visual labeled as "2" shows the distribution for individuals diagnosed with dementia. As can be seen from the figure, the diseases that occur just before receiving an AD/dementia diagnosis are organ failures and cerebrovascular events. Hypertension, on the other hand, appears approximately 3 years before the diagnosis. Additionally, the year just before the AD/dementia diagnosis is a period with a high likelihood of developing pre-diseases.

The prevalence of pre-diseases is highlighted in both presented figures. Accordingly, Fig. 3 presents the average time in months of how long pre-diseases appeared before the AD/dementia diagnosis process.

As can be understood from Fig. 3, hypertension, which has the highest morbidity, is also the earliest experienced disease for both groups. Similarly, among the top five diseases in terms of morbidity ranking, diabetes, hyperlipidemia, and psychological disorders are among the first five diseases experienced. Additionally, it is observed that organ failures and cerebrovascular events occur just before the diagnosis of AD/dementia.

After presenting graphical illustrations, our study includes logistic regression (LR) analysis to examine the impact of age, sex, and citizenship status on AD/dementia. Table 2 displays the logistic regression analysis outputs. From the table, it is evident that women face AD with a 10.5% higher risk than men, while this rate is 6.8% for dementia. The results also indicate that a one-point increase in the 'age' variable raises the risk of AD by 1.5% and dementia by 0.6%. In contrast, the marginal impact of age and sex on AD/dementia is overshadowed by foreign citizenship, which increases the risk of AD by approximately 70% and dementia by 89%. However, the models' prediction success is limited to approximately 50%.

**Table 2:** Coefficients of LR Models (%)*.

Independent variable	Dependent variable			
	ad		dementia	
	β (%)	Ac. (%)	β (%)	Ac. (%)
Sex^1^	10.5	52.82	6.8	51.24
Age	1.5		0.6	
Foreigner^1^	69.8		88.5	

*The coefficients presented are statistically significant at least at 95% significance level (*).

**“β” illustrates the coefficients for the variable. All coefficients turned exponentially and given as percentage. “Ac.” Illustrates accuracy level of the model as percentage.

While the prevalence rates of these diseases may provide important predictions, the main aim of the study is to gain a new understanding of the interrelationships between these diseases and their relevance to the context of AD/dementia. In this regard, it is essential to interpret the outputs from PCG data. Summary information about the data used for PCG analysis will be found in Annex 4.

**Figure 4:**
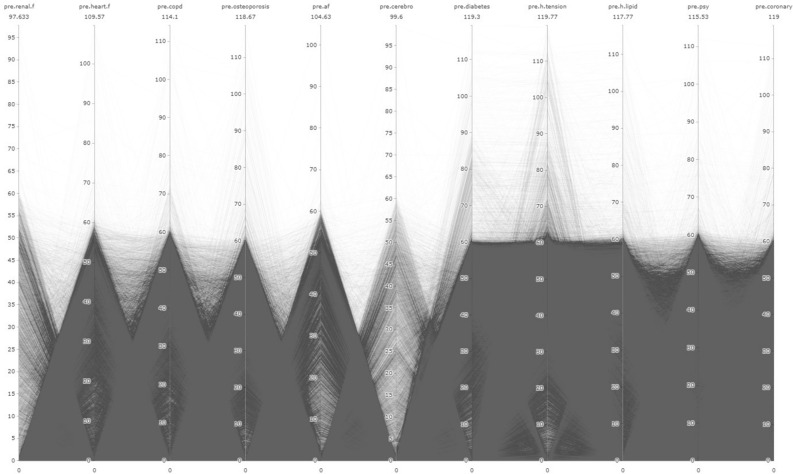
Relational analysis of diagnosed diseases in individuals with AD during the preclinical phase.

Figure 4 presents the PCG representation of pre-diseases for individuals diagnosed with AD. Additionally, visualizations for regions below and above the Turkey average are presented in Annex-5 and Annex-6, respectively. From these outputs, it can be understood that, in general, individuals diagnosed with AD have low renal.f density and a negative relationship between heart.f, ocpd, and osteoporosis. Furthermore, there is a strong correlation between hypertension and diabetes. Additionally, there is an inverse relationship between h.lipid, psy, and coronary.

**Figure 5:**
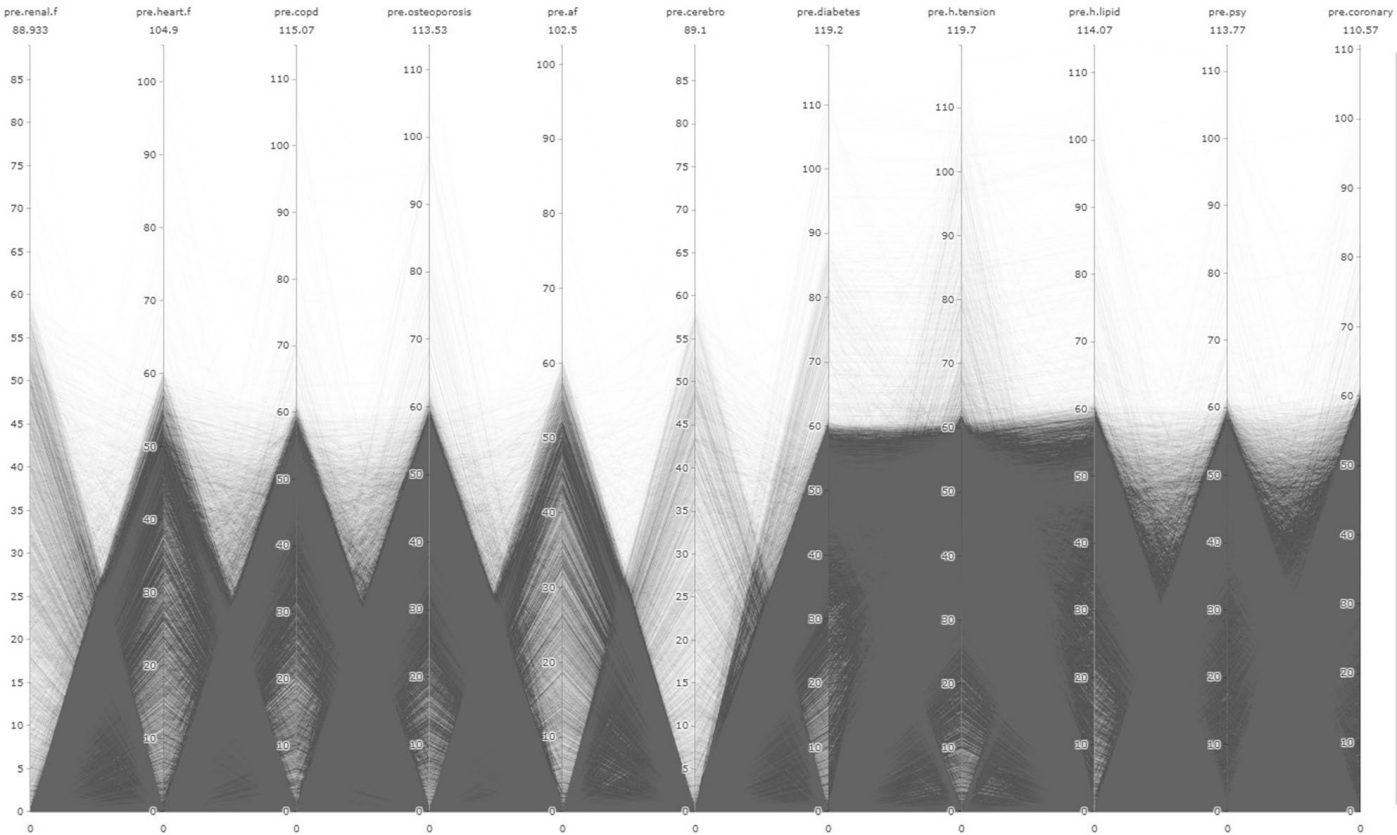
Relational analysis of diagnosed diseases in individuals with dementia during the preclinical phase.

Figure 5 presents the distribution of pre-diseases in individuals diagnosed with dementia. The distribution for regions with average values below and above the Turkey average is shown in Annex-7 and Annex-8, respectively. According to this, the density and distribution of pre-diseases are characteristic similar to the distribution of individuals diagnosed with AD. Additionally, it is noticeable that cerebrovascular density is low. Furthermore, the strongest correlation remains between hypertension and diabetes.

**Figure 6:**
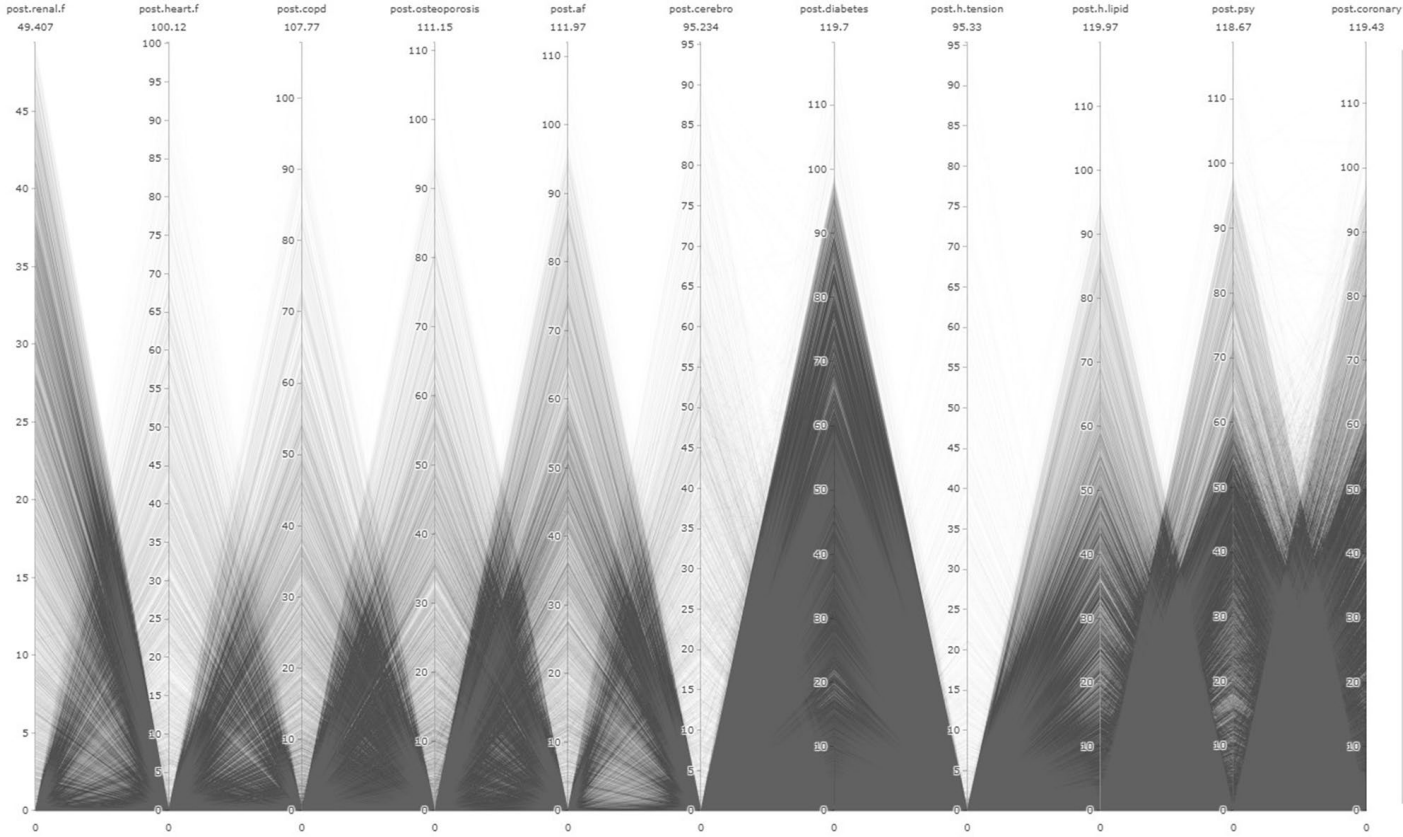
Relational analysis of diagnosed diseases in ındividuals with AD during the postclinical phase.

PCG outputs for individuals diagnosed with AD are presented in Fig. 6 for the entire dataset, and the distinction for regions above and below the Turkey average is provided in Annex-9 and Annex-10, respectively. In relation to this, h.tension, cerebro, and heart.f diseases have the lowest density. While there is a negative correlation among all diseases, diabetes and renal.f are significantly more experienced in a dense manner.

**Figure 7:**
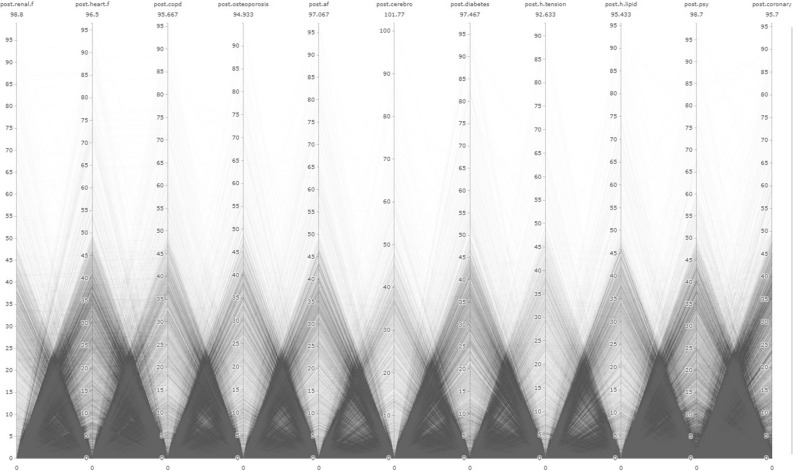
Relational analysis of diagnosed diseases in ındividuals with dementia during the postclinical phase.

The overall distribution of post-diseases for individuals diagnosed with dementia is presented in Fig. 7. Additionally, analyses for regions above and below the country average are provided in Annex-11 and Annex-12, respectively. Once again, there is a negative correlation among all diseases. However, in terms of specific diseases, cerebro and h.tension have low density, while psy, heart.f, af, diabetes, and coronary diseases have higher incidence rates.

## Discussion and conclusion

In recent years, significant advancements have been made in understanding the pathogenesis of Alzheimer's disease (AD) through the accumulation and monitoring of biomarkers, shedding light on the accumulation and prevalence of AD pathology. Blood biomarkers used to predict the pathology of AD are believed to form the basis for future-generation approaches, focusing on the detection and management of risk before the onset of neurodegenerative processes, and paving the way for proactive interventions.

Additionally, looking to the past is important to comprehend the present and gain insight into the future. Currently, the exaggerated expectations associated with the development of drug therapies for AD, targeting Aβ accumulation, pathological tau, and neurodegeneration (ATN classification system) with symptomatic-based pleiotropic effects, are reminiscent of the process in the 1990s. During that time, genetic-based Aβ definitions for AD diagnosis were established, followed by optimistic expectations for the development of effective therapeutic drugs, yet unfortunately, these expectations have not been met up to the present day. While these endeavors are valuable and serve as guiding principles, they underscore the necessity to approach AD from various perspectives and also prompt a reevaluation of the ineffectiveness of conventional approaches in yielding successful modifying therapeutic strategies.

To date, numerous scientific studies have explored the relationships between AD's characteristic features and other chronic diseases. However, a comprehensive understanding of the temporal connection between AD pathogenesis and chronic diseases in a holistic context has not been demonstrated. This study is focused on achieving this strategic objective.

A relatively recent discovery suggesting that Alzheimer's disease may start 20 years or more before the onset of symptoms indicates an important time frame for potential intervention in the disease's progression. Similarly, multiple studies have indicated that neuronal and synaptic loss in the brain occurs concurrently with the widespread development of tau pathology during the initial clinical stages of AD, revealing the presence of a complex pathology associated with long-term brain development^10–12^. In one study, cognitive impairments were found to begin 5 years before symptom appearance, yet it was understood that these patients only met the diagnostic criteria for AD 3 years after the onset of symptoms^11^. In another study, structural modifications in the brain were found to start 5 years before symptom manifestation^12^. Indeed, in the extensive dataset from Turkey, it was determined that 87.8% of individuals diagnosed with AD had received diagnoses of hypertension primarily around 60 months before the AD diagnosis (see Fig. 4). Hypertension is considered the first gateway to the pathology of AD, accompanied by coronary artery disease (average 18 months), diabetes (average 15 months), hyperlipidemia (average 12 months), and depression (average 12 months). Chronic diseases diagnosed during the preclinical phase manifest as structural impairments at the organ level, such as heart failure (average 6 months), COPD (average 8 months), osteoporosis (average 11 months), and kidney failure (average 2 months).

**Figure 8:**
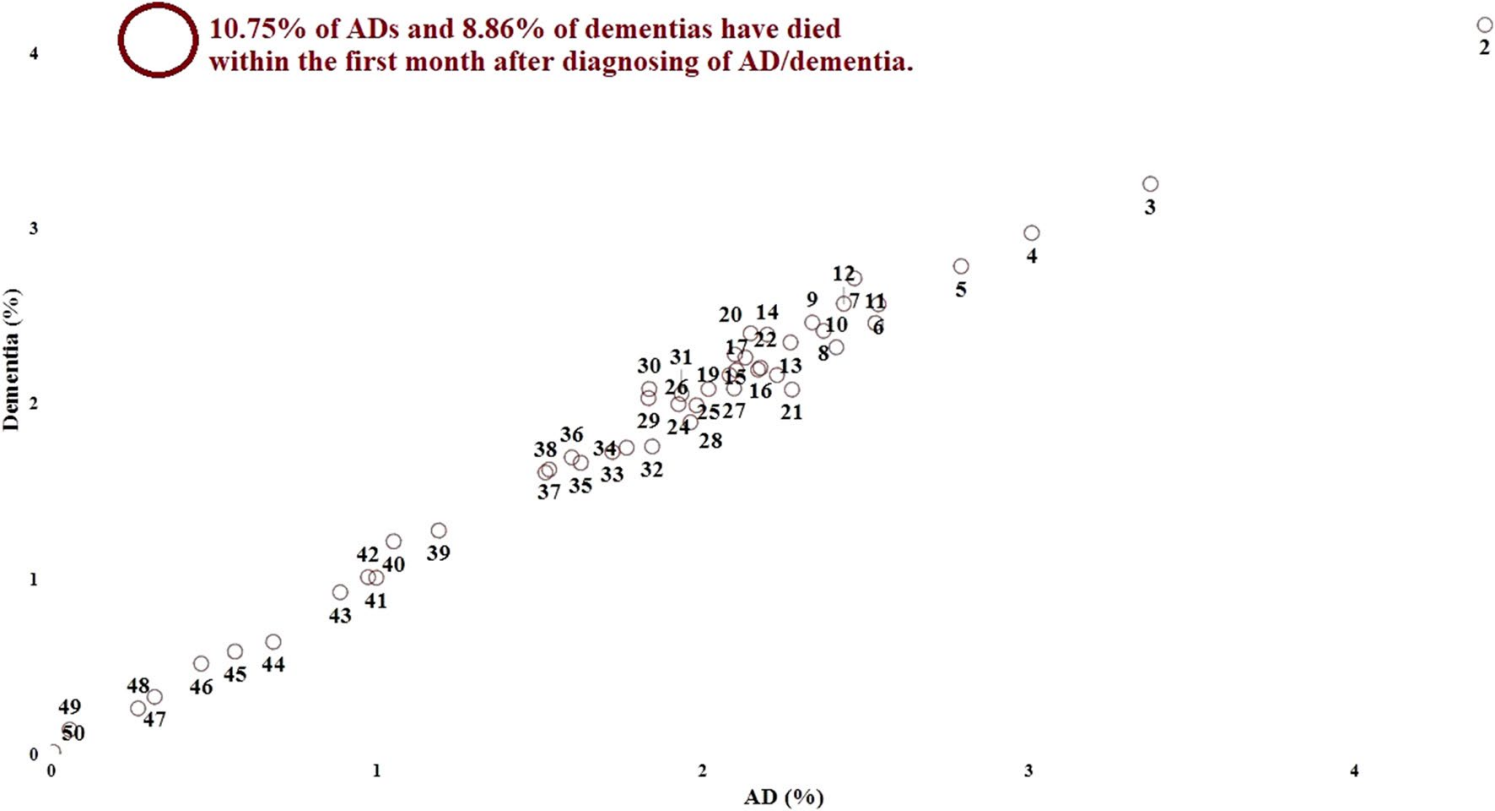
Distribution of post-diagnosis lifespan in ad/dementia (months). The figure presents the distribution of months after the diagnosis when individuals diagnosed with AD/dementia passed away. Among the individuals with available data in the dataset, 49.16% of those diagnosed with AD and 46.53% of those diagnosed with dementia had passed away as of February 2023. As evident from the figure, individuals with AD/dementia diagnoses who have passed away are predominantly within a close time frame to the diagnosis. In fact, 41.43% of those diagnosed with AD and 39.51% of those diagnosed with dementia had passed away within 12 months after diagnosis. This is supported by the negative correlation calculated at the level of (-0.7) between the total months survived post-diagnosis and AD, and (-0.77) between dementia. In other words, a decrease in the total months survived is observed among individuals diagnosed with AD.

In contrast to the preclinical phase, during the clinical phase, individuals with AD have been predominantly observed not to develop significant new diagnoses of chronic diseases. Even if a single new disease is developed among AD patients during the clinical phase (average 0.83), there is no apparent correlation with another disease. These findings are visualized in the Turkey average (TA) post general PCG visualization (see Fig. 6). Furthermore, individuals with AD during the clinical phase have relatively shorter average lifespans. These findings can be interpreted as a kind of terminal phase for patients with AD. Figure 8 displays the distribution of data related to this aspect.

All these findings obtained from the analysis of Turkey's extensive data underscore the necessity of considering numerous chronic diseases that have a direct connection to AD's pathogenesis during the preclinical phase. Therefore, for drugs targeted at having pleiotropic effects to truly be transformative, restoring just the functional brain circuits to their previous state will not be sufficient. These drugs need to modify the long-term damage caused by other chronic diseases, particularly the damage that develops over time and especially within the general vascular system. Moreover, these drugs should not only address these issues but also revert the basal cerebral hypometabolism to its former state. All these requirements, at best, correspond to an individual's general physiological state approximately 10 years before receiving an AD diagnosis. This is because various studies have shown that^10–12^ about 10 years before symptom onset, a deceleration in basal cerebral metabolism (cerebral hypometabolism) has been observed in the brain.

Because during the preclinical phase, chronic diseases and the complex interconnections between them can lead to significant functional losses in brain functionality, general physiology, and the vascular system. This functional loss progresses slowly at first but gradually gives way to an exponentially advancing process of deterioration with each new diagnosis of a chronic disease. The fact that late-stage disease diagnoses emerging during the preclinical process are obtained just before an AD diagnosis is the most significant indicator of this. This phenomenon is illustrated in the relevant connectivity mapping (see Fig. 4).

The preventability of AD primarily depends on not opening the first gate (hypertension) that initiates the process. After the first gate is opened, the occurrence of new diseases (additional gates) and the increasing complexity of interconnections between them make it more challenging to manage the risk. During these asymptomatic years, diagnoses related to various diseases, especially hypertension, lead to both cognitive function loss and structural modifications in the brain, accompanied by concurrent development and accumulation of biomarker pathology. On the other hand, even if a hypertension diagnosis is obtained, the focus of prophylactic intervention should be on disrupting the connection between hypertension and other diseases. Otherwise, at this stage, even if the current AD diagnostic criteria are not yet met, AD becomes inevitable. This is because during the preclinical process, when early changes in the path to AD start occurring, the brain compensates for them, allowing individuals to continue functioning normally. However, this process will not continue indefinitely. During the preclinical phase, the techniques used in cognitive, neurological, and behavioral evaluations, along with MRI, computed tomography (CT), brain imaging, and advanced methods used in cases of suspected diagnosis such as PET and cerebrospinal fluid (CSF) analysis, are far from having the resolution and explanatory power needed to detect the neuropathology that begins with the development of hypertension. Thus, it appears that the current diagnostic techniques can only detect AD pathology at an irreversible threshold, similar to the phenomena occurring in the singularity stage of matter before the formation of a black hole. Given the current methods and techniques, achieving such a stage's functional reversal seems unlikely. Therefore, while efforts directed towards developing a single drug with pleiotropic effects or a combination are valuable, it suggests that the characteristic nature of AD is not yet fully understood. This is because AD does not exhibit a disease characteristic that can be understood solely through symptomatic approaches.

It appears that the preventability of AD relies not on the threshold based on current symptomatic approaches but rather on effective and optimized prophylactic interventions occurring much earlier. The true preventability of AD involves achieving manageable risk along with the recognition of risk and breaking the patterns formed by interconnections between chronic diseases. Naturally, this perspective may also enable the monitoring and manageability of neurodegeneration caused by Aβ or tau. However, the crucial point to understand at this juncture is that molecular neurodegeneration arises as a consequence of gradual deterioration in the overall physiology and vascular system caused by chronic diseases, rather than being the direct causes of AD. Because these are not proteins we obtain from food. They are substances produced by the body itself, with an active role during a period, and since they cannot be broken down, they accumulate and cause toxic effects.

In summary, interventions based on symptomatic approaches targeting Aβ or tau-related neurodegeneration do not seem viable within the context of this study when evaluated for their potential to provide therapeutic modification in AD. The deposition and dissemination at the molecular neurodegenerative level are outcomes resulting from the gradual deterioration caused by chronic diseases within the general physiology, metabolism, and vascular system. Therefore, AD can be considered not as an independent disease caused solely by causal factors, but rather as the final stage reached after a prolonged and complex process.

### Supplementary Information

Supplementary Information 1.

Supplementary Information 2.

Supplementary Information 3.

Supplementary Information 4.

Supplementary Information 5.

Supplementary Information 6.

Supplementary Information 7.

Supplementary Information 8.

Supplementary Information 9.

## Data Availability

The data that support the findings of this study are available from the Ministry of Health of the Republic of Turkey but restrictions apply to the availability of these data, which were used under license for the current study, and so are not publicly available. Data are however available from the authors upon reasonable request and with permission of the Ministry of Health of the Republic of Turkey. Someone who wants to request the data should get in contact with Assoc. Prof. Dr. Talip Yiğit the corresponding author of the study.
