# Supplementary material for: Insights from Turkey's big data: unraveling the preventability, pathogenesis, and risk management of Alzheimer's disease (AD)
Source: Sci Rep. 2024 Mar 12;14:6005. doi: 10.1038/s41598-024-56702-1 (PMC10933367; doi:10.1038/s41598-024-56702-1)
Supplement: Supplementary file 1 — Supplementary Information 1. [file 41598_2024_56702_MOESM1_ESM.docx]

| Variable  Group | Name | Type  [Observation:  ad / dementia]* | Explanation |
| --- | --- | --- | --- |
| Dependent Variables | ad | Binary  **[0:** 87.536**]**  **[1:** 87.511**]** | Whether the person has been diagnosed with Alzheimer's  *Only in the "alzheimer's" dataset  (ICD 10 Codes: F00.0, F00.1, F00.2, F00.9 or G30) |
|  | dementia | Binary  **[0:** 62.155**]**  **[1:** 62.139**]** | Whether the person has been diagnosed with Dementia  *Only in the "dementia" dataset  (ICD 10 Codes: F01, F02, F02.0, F02.1, F02.2, F02.3, F02.4, F02.8, F03 or G31 ) |
| Independent Variables | sex | Binary  **[0:** 68.655 / 49.402**]**  **[1:** 106.392 / 74.892**]** | Biological sex of the person (0: Male 1: Female) |
|  | age | Continuous  **[Min:** 65 / 65 **]**  **[Mean:** 79.23 / 78.96 **]**  **[Max:** 103 / 103 **]** | Age of the person |
|  | foreigner | Binary  **[0:** 174.908 / 124.212**]**  **[1:** 139 / 82**]** | Whether the person has citizenship of  the Republic of Türkiye  (0: Yes 1: No) |

**Appendix 1.: Explanations on the Datasets**

*After the outlier analysis conducted for continuous “age” variable.
