# Supplementary material for: Insights from Turkey's big data: unraveling the preventability, pathogenesis, and risk management of Alzheimer's disease (AD)
Source: Sci Rep. 2024 Mar 12;14:6005. doi: 10.1038/s41598-024-56702-1 (PMC10933367; doi:10.1038/s41598-024-56702-1)
Supplement: Supplementary file 2 — Supplementary Information 2. [file 41598_2024_56702_MOESM2_ESM.docx]

**Appendix-1.1: R Studio Codes for the Logistic Regression Analysis**

#1.Intalling required packages

install.packages("readxl")

install.packages("Rcpp")

install.packages("tidyverse")

install.packages("caret")

install.packages("stats")

install.packages("caTools")

install.packages("psych")

library(readxl)

library(Rcpp)

library(tidyverse)

library(caret)

library(stats)

library(caTools)

library(psych)

#Uploading and Shaping the Data

df <- dementia_logisticregression[,-1]

df$ad <- as.factor(df$ad)

df$dementia <- as.factor(df$dementia)

df$sex <- as.factor(df$sex)

df$foreigner <- as.factor(df$foreigner)

summary(df)

#Outlier Analysis for the Countinuos Variable

outliers <- function(x) {

Q1 <- quantile(x, probs=.25)

Q3 <- quantile(x, probs=.75)

iqr = Q3-Q1

upper_limit = Q3 + (iqr*1.5)

lower_limit = Q1 - (iqr*1.5)

x > upper_limit | x < lower_limit

}

remove_outliers <- function(df, cols = names(df)) {

for (col in cols) {

df <- df[!outliers(df[[col]]),]

}

df

}

summary(df)

df_cleaned <- remove_outliers(df, c('age'))

summary(df_cleaned)

str(df_cleaned)

df <- df_cleaned

#Separating the Data Set as Test and Train Data

set.seed(123)

df_2 = sort(sample(nrow(df), nrow(df)*.8))

df_train<- df[df_2,]

df_test<- df[-df_2,]

summary(df_train)

summary(df_test)

#Building the Model and Prediction

lr.model <- glm(dementia ~ relevel(sex,ref="0")+

age+

relevel(foreigner,ref="0"),

data=df_train, family="binomial")

summary(lr.model)

pred <- predict(lr.model,df_test, type="response")

pred <- as.integer(pred>0.5)

confusionMatrix(as.factor(pred),df_test$dementia)

#Exponential Transformation of Coefficient

(exp(lr.model$coefficients[-1])-1)
