# Supplementary material for: Insights from Turkey's big data: unraveling the preventability, pathogenesis, and risk management of Alzheimer's disease (AD)
Source: Sci Rep. 2024 Mar 12;14:6005. doi: 10.1038/s41598-024-56702-1 (PMC10933367; doi:10.1038/s41598-024-56702-1)
Supplement: Supplementary file 3 — Supplementary Information 3. [file 41598_2024_56702_MOESM3_ESM.docx]

**Appendix- 2.: Logistic Regression Models’ Detailed Outputs**

**Logistic Regression Models’ Outputs for “ad” Dependent Variable**

| **Dependent Variable: ‘ad’ (0-1)** | | | | | |
| --- | --- | --- | --- | --- | --- |
| **Variable** | **Coefficient (β)** | **Standard Deviation** | **z Value** | **p Value** | **Exponential Coefficient (β)** |
| **Logistic Regression Model Built with Training and Test Data (%70 - %30)** | | | | | |
| **Intercept** | -1.2640976 | 0.0601974 | -20.999 | <2e-16*** | --- |
| **sex (1)** | 0.0998189 | 0.0117665 | 8.483 | <2e-16*** | 0.10497074 |
| **age** | 0.0152073 | 0.0007585 | 20.050 | <2e-16*** | 0.01532347 |
| **foreigner (1)** | 0.4766594 | 0.2133399 | 2.234 | 0.0255* | 0.61068471 |
| **Logistic Regression Model Built with Training and Test Data (%80 - %20)** | | | | | |
| **Intercept** | -1.2352367 | 0.0563259 | -21.930 | < 2e-16*** | --- |
| **sex (1)** | 0.1000475 | 0.0110073 | 9.089 | < 2e-16*** | 0.10522344 |
| **age** | 0.0148115 | 0.0007096 | 20.873 | < 2e-16*** | 0.01492178 |
| **foreigner (1)** | 0.5296025 | 0.2002282 | 2.645 | 0.00817** | 0.69825704 |

**Significancy Levels: 0 ‘***’ 0.001 ‘**’ 0.01 ‘*’ 0.05 ‘.’ 0.1 ‘ ’ 1**

**Confusion Matrix of Logistic Regression Models for “ad” Dependent Variable**

| **Dependent Variable: ‘ad’ (0-1)** | | | | |
| --- | --- | --- | --- | --- |
| **Value** | **Train %70 – Test %30** | | **Train %80 – Test %20** | |
|  | **0** | **1** | **0** | **1** |
| **0** | 13812 | 12352 | 9256 | 8264 |
| **1** | 12515 | 13836 | 8252 | 9238 |
| **AIC Value** | **169360** | | 193577 | |
| **Accuracy** | 0.5265 | | **0.5282** | |
| **Sensitivity** | 0.5246 | | **0.5287** | |
| **Specificity** | **0.5283** | | 0.5278 | |

**Logistic Regression Models’ Outputs for “dementia” Dependent Variable**

| **Dependent Variable: ‘dementia’ (0-1)** | | | | | |
| --- | --- | --- | --- | --- | --- |
| **Variable** | **Coefficient (β)** | **Standard Deviation** | **z Value** | **p Value** | **Exponential Coefficient (β)** |
| **Logistic Regression Model Built with Training and Test Data (%70 - %30)** | | | | | |
| **Intercept** | -0.4972185 | 0.0706134 | -7.041 | 1.90e-12*** | --- |
| **sex (1)** | 0.0662383 | 0.0139183 | 4.759 | 1.94e-06*** | 0.068481336 |
| **age** | 0.0057588 | 0.0008935 | 6.446 | 1.15e-10*** | 0.005775441 |
| **foreigner (1)** | 0.6340942 | 0.2776634 | 2.284 | 0.0224* | 0.885313615 |
| **Logistic Regression Model Built with Training and Test Data (%80 - %20)** | | | | | |
| **Intercept** | -0.5243483 | 0.0659922 | -7.946 | 1.93e-15*** | --- |
| **sex (1)** | 0.0625987 | 0.0130160 | 4.809 | 1.51e-06*** | 0.06459951 |
| **age** | 0.0061746 | 0.0008346 | 7.398 | 1.38e-13*** | 0.00619374 |
| **foreigner (1)** | 0.6071050 | 0.2495637 | 2.433 | 0.015* | 0.83511111 |

**Significancy Levels: 0 ‘***’ 0.001 ‘**’ 0.01 ‘*’ 0.05 ‘.’ 0.1 ‘ ’ 1**

**Confusion Matrix of Logistic Regression Models for “dementia” Dependent Variable**

| **Dependent Variable: ‘dementia’ (0-1)** | | | | |
| --- | --- | --- | --- | --- |
| **Value** | **Train %70 – Test %30** | | **Train %80 – Test %20** | |
|  | **0** | **1** | **0** | **1** |
| **0** | 9823 | 9403 | 6115 | 5845 |
| **1** | 8780 | 9283 | 6356 | 6543 |
| **AIC Value** | **120547** | | 137764 | |
| **Accuracy** | **0.5124** | | 0.5092 | |
| **Sensitivity** | **0.5280** | | 0.4903 | |
| **Specificity** | 0.4968 | | **0.5282** | |
