# Supplementary material for: Insights from Turkey's big data: unraveling the preventability, pathogenesis, and risk management of Alzheimer's disease (AD)
Source: Sci Rep. 2024 Mar 12;14:6005. doi: 10.1038/s41598-024-56702-1 (PMC10933367; doi:10.1038/s41598-024-56702-1)
Supplement: Supplementary file 4 — Supplementary Information 4. [file 41598_2024_56702_MOESM4_ESM.docx]

**Annex-4**

The Parallel Coordinate Graph method is used to display the relationship between multiple variables in two-dimensional space. The method allows the relationship between units (variables) to be displayed on parallel axes. Thus, the relationship between more than two variables can be examined. In this way, it can be observed how different factors related to a situation arise together. In terms of our study, this presented important findings regarding the co-occurrence of different diseases in the context of AD/D. The proportional distribution and descriptive statistics for these diseases are presented below.

**Proportional Distributions of “Sex” and “Age Groups”**

**for the Data Used in Parallel Coordinats Graphs**

| **Sex / Age** | **Data for Before Alzheimers Diagnosing** | **Data for Before Dementia Diagnosing** | **Data for After Alzheimers Diagnosing** | **Data for After Dementia Diagnosing** |
| --- | --- | --- | --- | --- |
| **Sex: Male (%)** | 37.74% | 38.79% | 37.73% | 38.73% |
| **Sex: Female (%)** | 62.26% | 61.21% | 62.27% | 61.27% |
| **Age:** (1)65-69 | 11.00% | 12.69% | 11.01% | 12.68% |
| **Age:** (2)70-74 | 16.50% | 17.86% | 16.50% | 17.89% |
| **Age:** (3)75-79 | 21.29% | 21.24% | 21.28% | 21.24% |
| **Age:** (4)80-84 | 21.40% | 20.62% | 21.39% | 20.63% |
| **Age:** (5)85-89 | 19.42% | 17.88% | 19.42% | 17.88% |
| **Age:** (6)90-94 | 8.20% | 7.79% | 8.19% | 7.76% |
| **Age:** (7)95-99 | 1.86% | 1.61% | 1.86% | 1.61% |
| **Age:** (8)100+ | 0.34% | 0.31% | 0.33% | 0.31% |

**Proportional Distributions of Ilnnesses Experienced Before AD/D Diagnosing**

**for the Data Used in Parallel Coordinate Graphs**

| **Illness** | **Alzheimers Dataset** | | **Dementias Dataset** | |
| --- | --- | --- | --- | --- |
|  | **Diagnosed (%)** | **Undiagnosed (%)** | **Diagnosed (%)** | **Undiagnosed (%)** |
| **pre.renal.f** | 9.88 | 90.12 | 8.94 | 91.06 |
| **pre.heart.f** | 22.67 | 77.33 | 20.18 | 79.82 |
| **pre.copd** | 27.57 | 72.43 | 26.02 | 73.98 |
| **pre.osteoporosis** | 33.66 | 66.34 | 32.2 | 67.8 |
| **pre.af** | 16.35 | 83.65 | 14.75 | 85.25 |
| **pre.cerebro** | 10.41 | 89.59 | 8.2 | 91.80 |
| **pre.diabetes** | 42.44 | 57.56 | 40.03 | 59.97 |
| **pre.h.tension** | 87.74 | 12.26 | 84.89 | 15.11 |
| **pre.h.lipid** | 37.93 | 62.07 | 35.16 | 64.84 |
| **pre.psy** | 45.36 | 54.64 | 40.72 | 59.28 |
| **pre.coronary** | 55.09 | 44.91 | 50.71 | 49.29 |

**Proportional Distributions of Ilnnesses Experienced After AD/D Diagnosing**

**for the Data Used in Parallel Coordinate Graphs**

| **Illness** | **Alzheimers Dataset** | | **Dementias Dataset** | |
| --- | --- | --- | --- | --- |
|  | **Diagnosed (%)** | **Undiagnosed (%)** | **Diagnosed (%)** | **Undiagnosed (%)** |
| **post.renal.f** | 6.65 | 93.35 | 7.19 | 92.81 |
| **post.heart.f** | 3.24 | 96.76 | 11.58 | 88.42 |
| **post.copd** | 6.3 | 93.70 | 8.61 | 91.39 |
| **post.osteoporosis** | 6.1 | 93.90 | 7.64 | 92.36 |
| **post.af** | 6.22 | 93.78 | 7.98 | 92.02 |
| **post.cerebro** | 2.63 | 97.37 | 5.89 | 94.11 |
| **post.diabetes** | 29.8 | 70.2 | 9.39 | 90.61 |
| **post.h.tension** | 1.3 | 98.70 | 6.49 | 93.51 |
| **post.h.lipid** | 17.02 | 82.98 | 7.78 | 92.22 |
| **post.psy** | 16.59 | 83.41 | 15.02 | 84.98 |
| **post.coronary** | 20.92 | 79.08 | 13.92 | 86.08 |

**Descriptive Statistics of the Data Used in Parallel Coordinats Graphs**

| **1.Descriptive Statistics of Illnesses Experienced Before Alzheimers Diagnosing** | | | | | | | | | | | |
| --- | --- | --- | --- | --- | --- | --- | --- | --- | --- | --- | --- |
| **Statistics** | **pre.renal.f** | **pre.heart.f** | **pre.copd** | **pre.osteoporosis** | **pre.af** | **pre.cerebro** | **pre.diabetes** | **pre.h.tension** | **pre.h.lipid** | **pre.psy** | **pre.coronary** |
| **Mean** | 2.33 | 6.09 | 8.81 | 11.67 | 4.28 | 2.07 | 16.5 | 39.39 | 13.58 | 12.88 | 18.84 |
| **Standard Deviation** | 8.91 | 14.06 | 17.21 | 19.2 | 12.05 | 8.31 | 22.52 | 20.17 | 20.46 | 19.11 | 21.34 |
| **Minimum Value** | 0 | 0 | 0 | 0 | 0 | 0 | 0 | 0 | 0 | 0 | 0 |
| **Maximum Value** | 97.63 | 109.57 | 114.1 | 118.67 | 104.63 | 99.6 | 119.3 | 119.77 | 117.77 | 115.53 | 119 |
| **Skewness Coefficient** | 4.28 | 2.32 | 1.77 | 1.34 | 2.96 | 4.57 | 0.88 | -0.85 | 1.13 | 1.18 | 0.59 |
| **Kurtosis Coefficient** | 18.51 | 4.32 | 1.74 | 0.34 | 7.79 | 21.47 | -0.78 | -0.09 | -0.24 | -0.04 | -1.13 |

| **2.Descriptive Statistics of Illnesses Experienced Before Dementia Diagnosing** | | | | | | | | | | | | | | | | |
| --- | --- | --- | --- | --- | --- | --- | --- | --- | --- | --- | --- | --- | --- | --- | --- | --- |
| **Statistics** | **pre.renal.f** | **pre.heart.f** | **pre.copd** | **pre.osteoporosis** | **pre.af** | **pre.cerebro** | | **pre.diabetes** | | **pre.h.tension** | | **pre.h.lipid** | | **pre.psy** | | **pre.coronary** |
| **Mean** | 2.11 | 5.27 | 8.1 | 10.58 | 3.73 | 1.5 | | 14.91 | | 35.74 | | 11.95 | | 11.14 | | 16.58 |
| **Standard Deviation** | 8.51 | 13.07 | 16.46 | 18.21 | 11.26 | 7.06 | | 21.56 | | 21.01 | | 19.3 | | 18.09 | | 20.43 |
| **Minimum Value** | 0 | 0 | 0 | 0 | 0 | 0 | | 0 | | 0 | | 0 | | 0 | | 0 |
| **Maximum Value** | 88.93 | 104.9 | 115.07 | 113.53 | 102.5 | 89.1 | | 119.2 | | 119.7 | | 114.07 | | 113.77 | | 110.57 |
| **Skewness Coefficient** | 4.49 | 2.56 | 1.89 | 1.47 | 3.23 | 5.44 | | 1.03 | | -0.58 | | 1.31 | | 1.39 | | 0.77 |
| **Kurtosis Coefficient** | 20.34 | 5.58 | 2.24 | 0.77 | 9.64 | 30.73 | | -0.45 | | -0.75 | | 0.27 | | 0.56 | | -0.88 |
| **3.Descriptive Statistics of Illnesses Experienced After Alzheimers Diagnosing** | | | | | | | | | | | | | | | | |
| **Statistics** | **post.renal.f** | **post.heart.f** | **post.copd** | **post.osteoporosis** | **post.af** | | **post.cerebro** | | **post.diabetes** | | **post.h.tension** | | **post.h.lipid** | | **post.psy** | **post.coronary** |
| **Mean** | 1.2 | 0.75 | 7.33 | 1.54 | 2.14 | | 0.68 | | 8.3 | | 0.16 | | 3.53 | | 4.69 | 5.44 |
| **Standard Deviation** | 5.7 | 5.51 | 7.33 | 8 | 10.22 | | 5.32 | | 18.64 | | 2.19 | | 11.45 | | 13.76 | 14.69 |
| **Minimum Value** | 0 | 0 | 0 | 0 | 0 | | 0 | | 0 | | 0 | | 0 | | 0 | 0 |
| **Maximum Value** | 49.41 | 100.12 | 107.77 | 111.15 | 111.97 | | 95.23 | | 119.7 | | 95.33 | | 119.97 | | 118.67 | 119.43 |
| **Skewness Coefficient** | 5.37 | 9.23 | 6.78 | 6.49 | 5.57 | | 9.47 | | 2.55 | | 20.34 | | 4.14 | | 3.44 | 3.27 |
| **Kurtosis Coefficient** | 29.63 | 96.57 | 52.32 | 47.35 | 33.23 | | 100.76 | | 6.05 | | 510.53 | | 19.11 | | 12.45 | 11.27 |

| **4.Descriptive Statistics of Illnesses Experienced After Dementia Diagnosing** | | | | | | | | | | | |
| --- | --- | --- | --- | --- | --- | --- | --- | --- | --- | --- | --- |
| **Statistics** | **post.renal.f** | **post.heart.f** | **post.copd** | **post.osteoporosis** | **post.af** | **post.cerebro** | **post.diabetes** | **post.h.tension** | **post.h.lipid** | **post.psy** | **post.coronary** |
| **Mean** | 1.48 | 2.38 | 1.72 | 1.52 | 1.66 | 1.13 | 1.84 | 1.06 | 1.54 | 2.27 | 2.62 |
| **Standard Deviation** | 6.95 | 8.59 | 7.45 | 6.91 | 7.37 | 5.96 | 7.6 | 5.54 | 6.98 | 7.99 | 8.83 |
| **Minimum Value** | 0 | 0 | 0 | 0 | 0 | 0 | 0 | 0 | 0 | 0 | 0 |
| **Maximum Value** | 98.8 | 96.5 | 95.67 | 94.93 | 97.07 | 101.77 | 97.47 | 92.63 | 95.43 | 98.7 | 95.7 |
| **Skewness Coefficient** | 5.77 | 4.45 | 5.38 | 5.61 | 5.46 | 6.5 | 5.12 | 6.72 | 5.54 | 4.49 | 4.14 |
| **Kurtosis Coefficient** | 38.63 | 22.73 | 33.36 | 36.04 | 34.42 | 48.54 | 30.49 | 53.09 | 35.07 | 23.01 | 19.4 |
