# Supplementary material for: Insights from Turkey's big data: unraveling the preventability, pathogenesis, and risk management of Alzheimer's disease (AD)
Source: Sci Rep. 2024 Mar 12;14:6005. doi: 10.1038/s41598-024-56702-1 (PMC10933367; doi:10.1038/s41598-024-56702-1)
Supplement: Supplementary file 5 — Supplementary Information 5. [file 41598_2024_56702_MOESM5_ESM.docx]

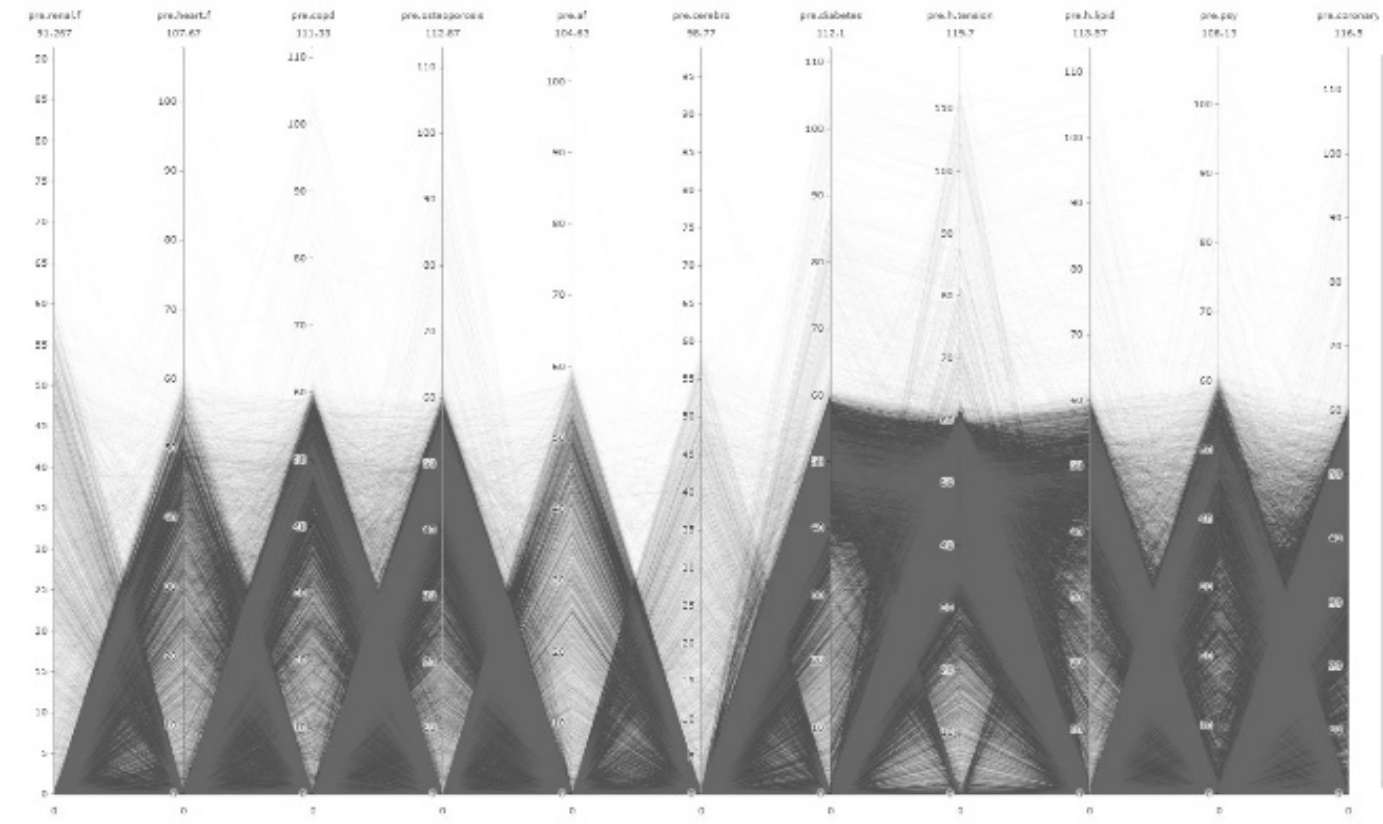


**Annex 5: Relational Analysis of Diagnosed Diseases of Individuals Diagnosed with AD in the Preclinical Process (In Regions Have Lower AD Rates than National Average)**


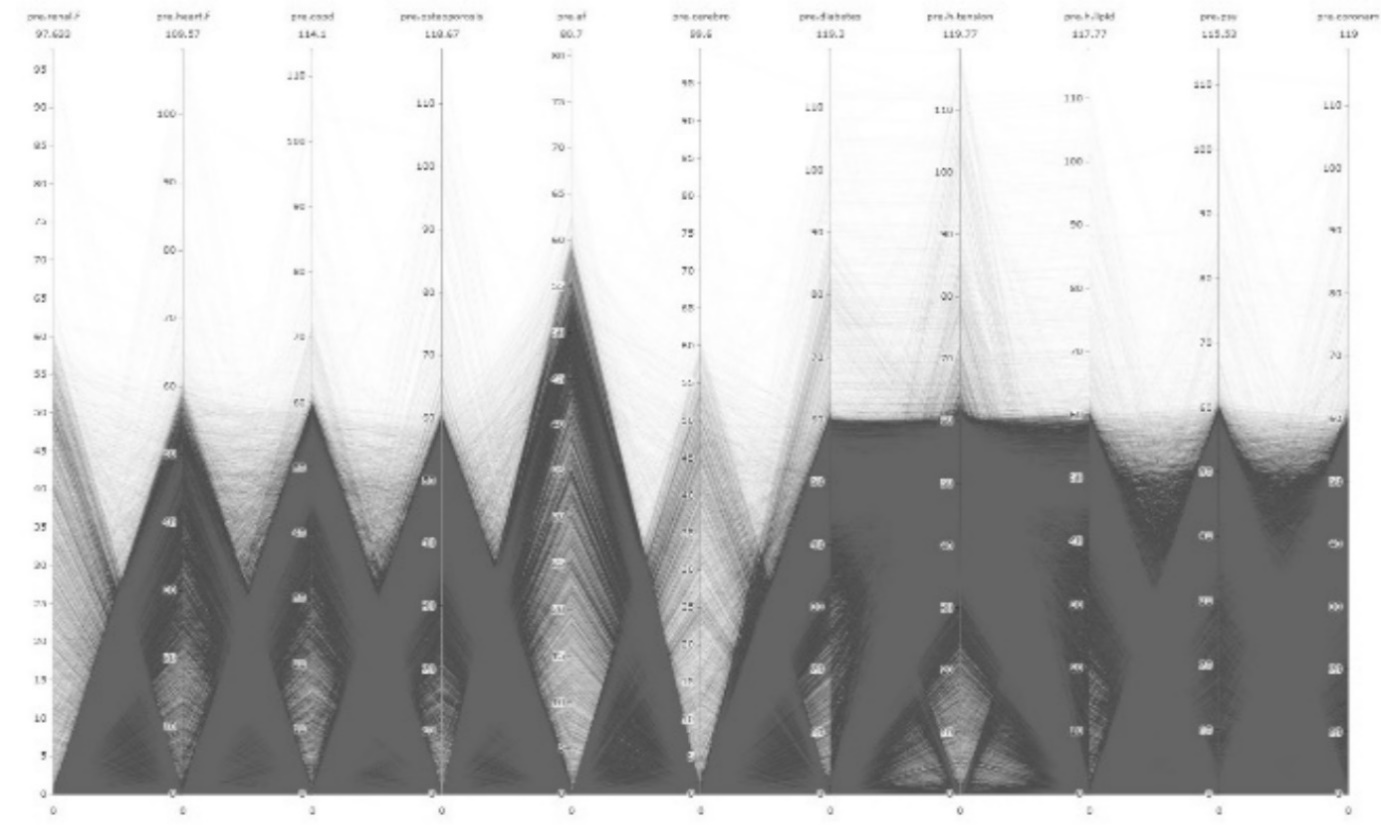


**Annex 6: Relational Analysis of Diagnosed Diseases of Individuals Diagnosed with AD in the Preclinical Process (In Regions Have Higher AD Rates than National Average)**


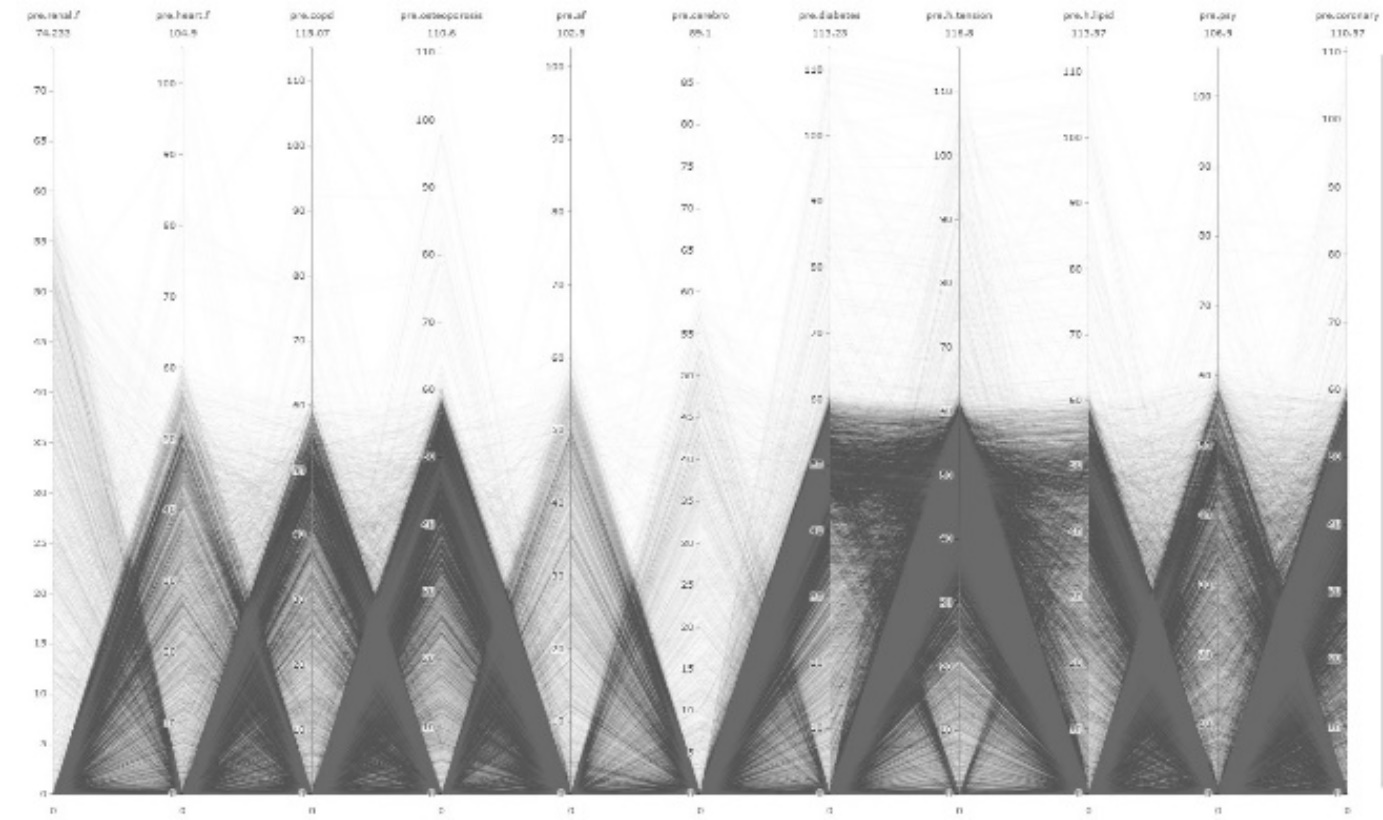


**Annex 7: Relational Analysis of Diagnosed Diseases of Individuals Diagnosed with Dementia in the Preclinical Process (In Regions Have Lower Dementia Rates than National Average)**


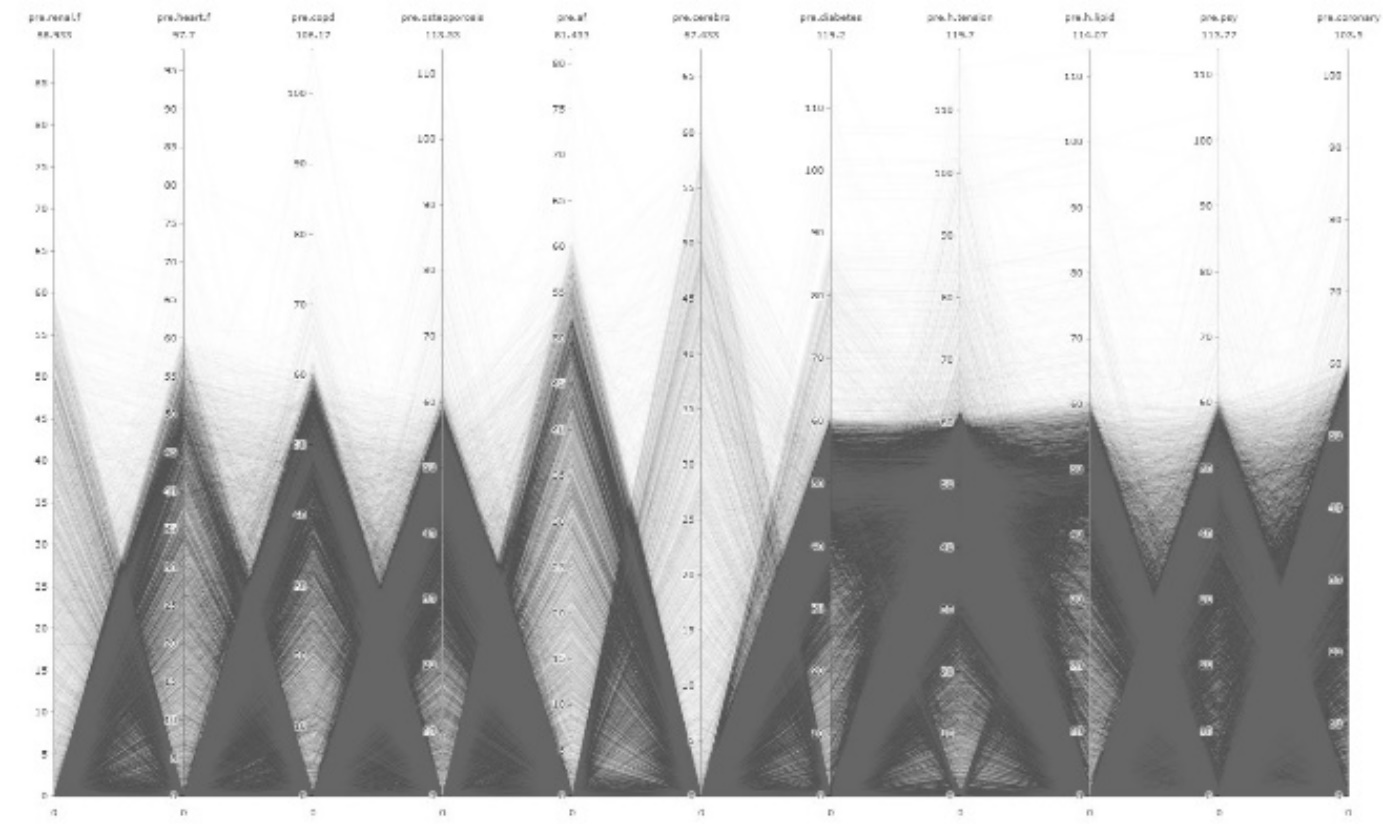


**Annex 8: Relational Analysis of Diagnosed Diseases of Individuals Diagnosed with Dementia in the Preclinical Process (In Regions Have Higher Dementia Rates than National Average)**

**
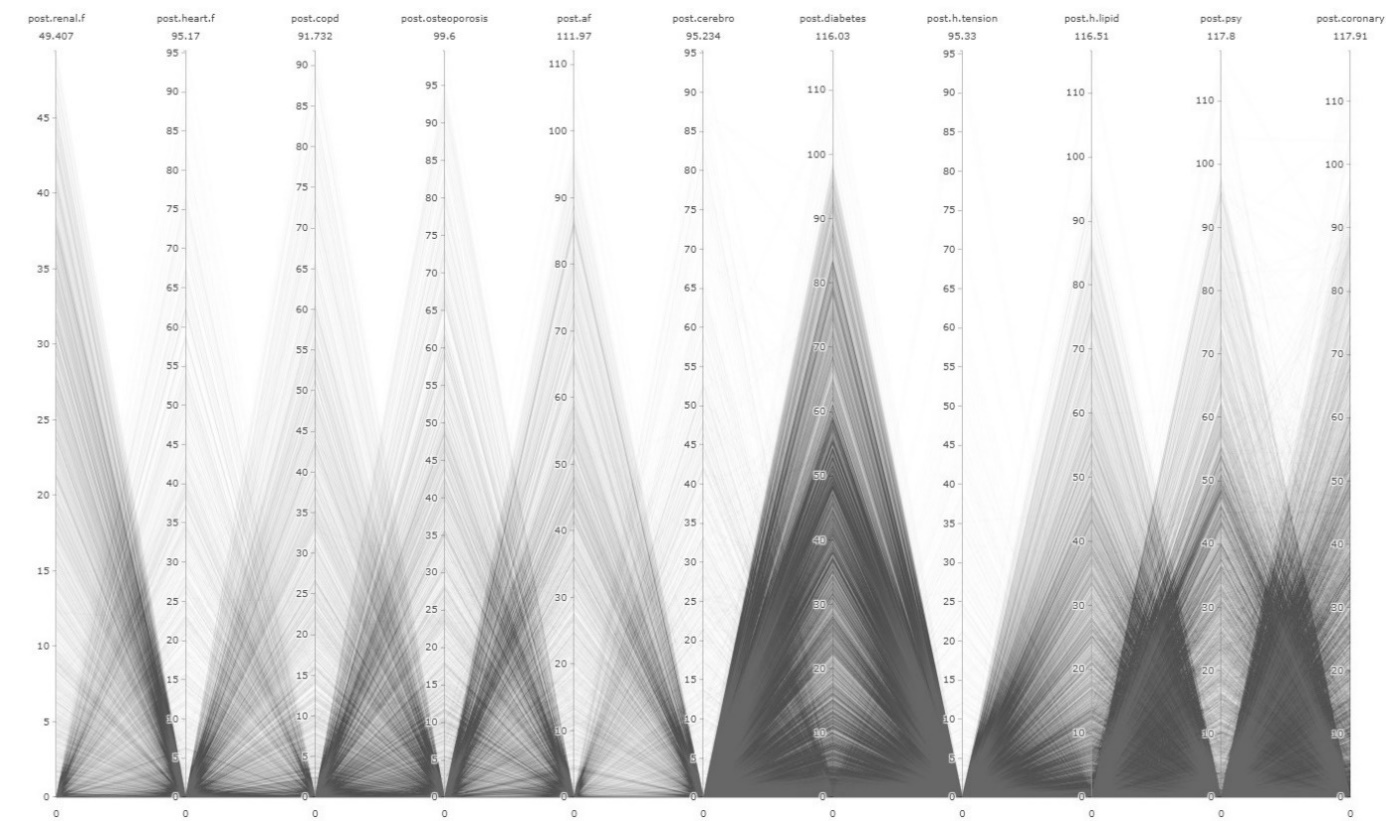
**

**Annex 9: Relational Analysis of Diagnosed Diseases of Individuals Diagnosed with AD in the Postclinical Process (In Regions Have Lower AD Rates than National Average)**


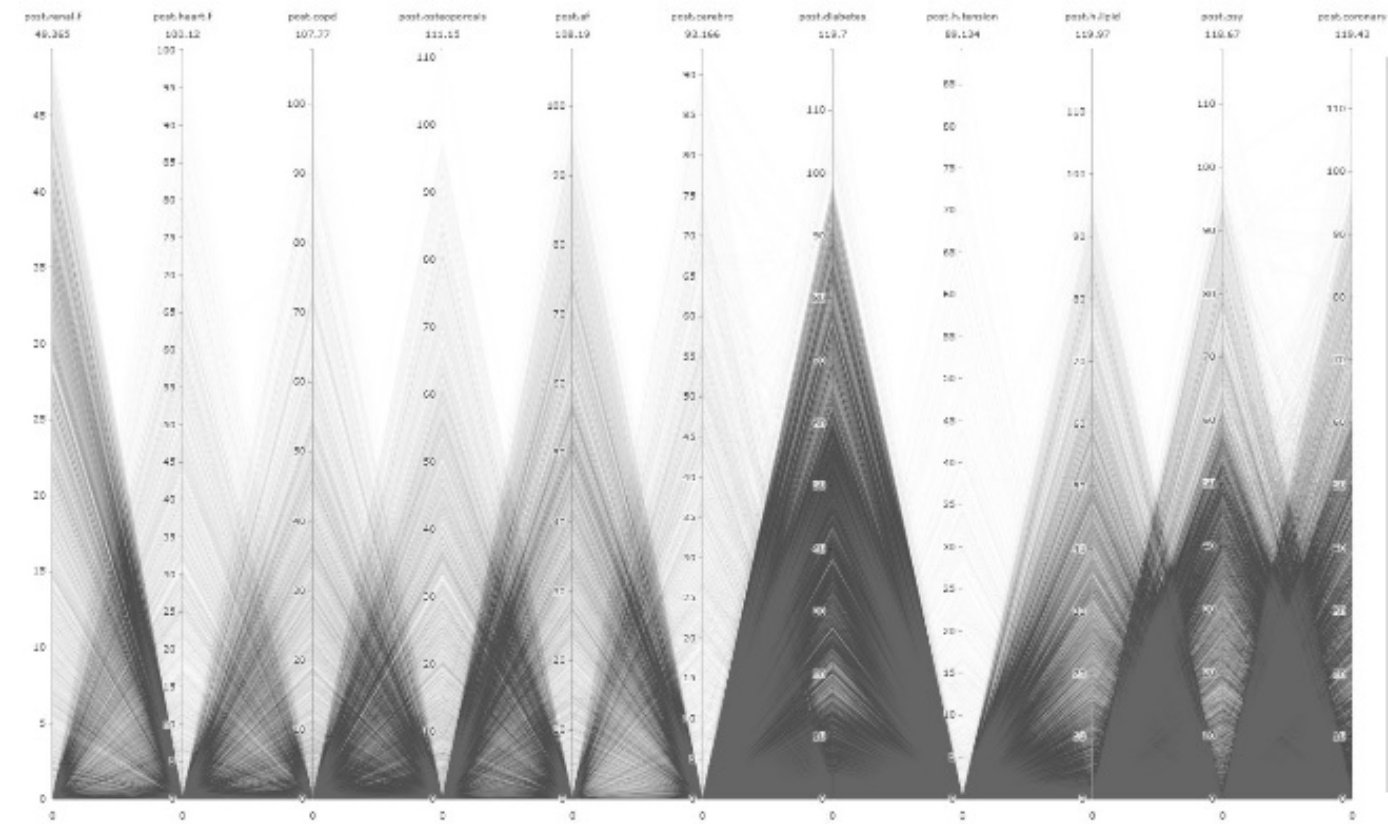


**Annex 10: Relational Analysis of Diagnosed Diseases of Individuals Diagnosed with AD in the Postclinical Process (In Regions Have Higher AD Rates than National Average)**


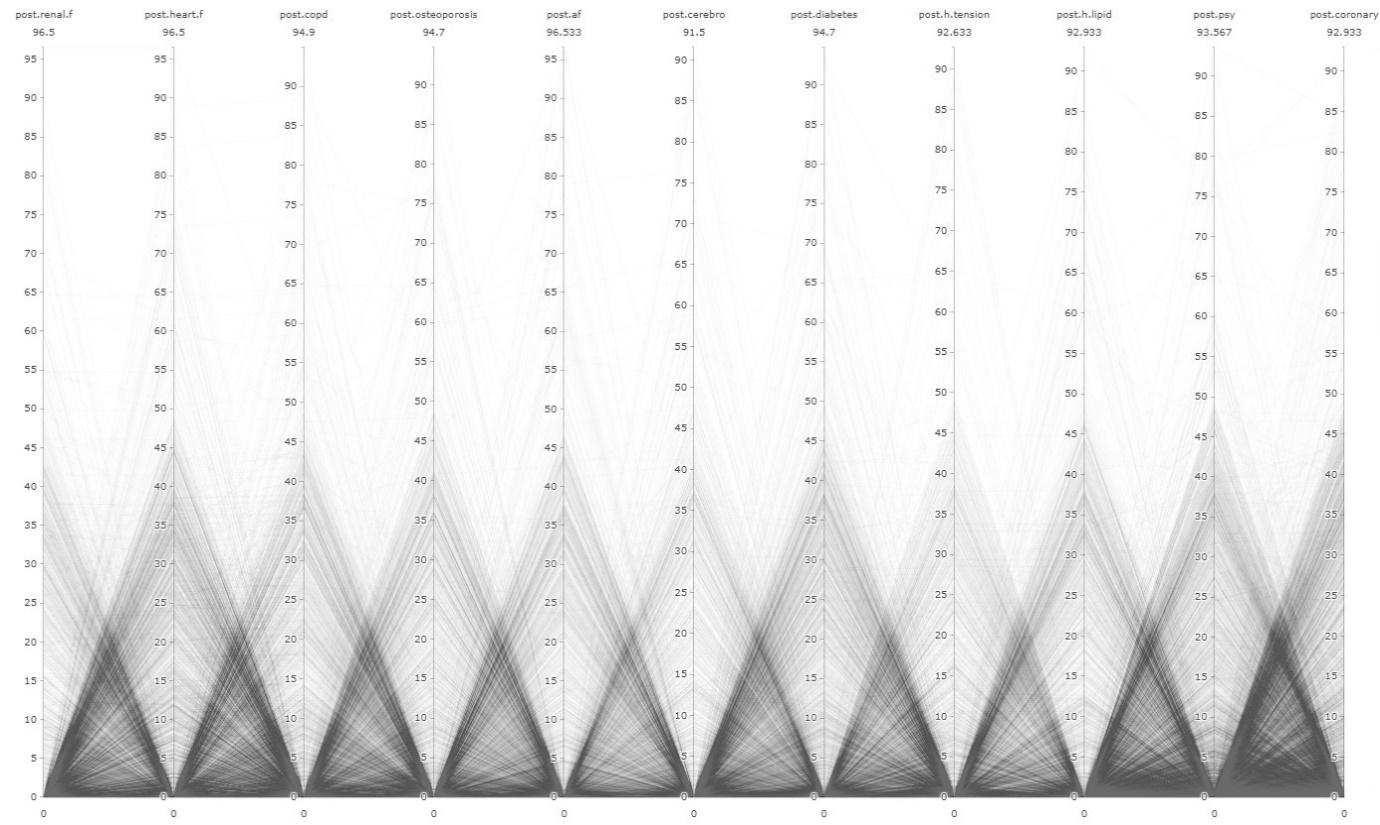


**Annex 11: Relational Analysis of Diagnosed Diseases of Individuals Diagnosed with Dementia in the Postclinical Process (In Regions Have Lower Dementia Rates than National Average)**


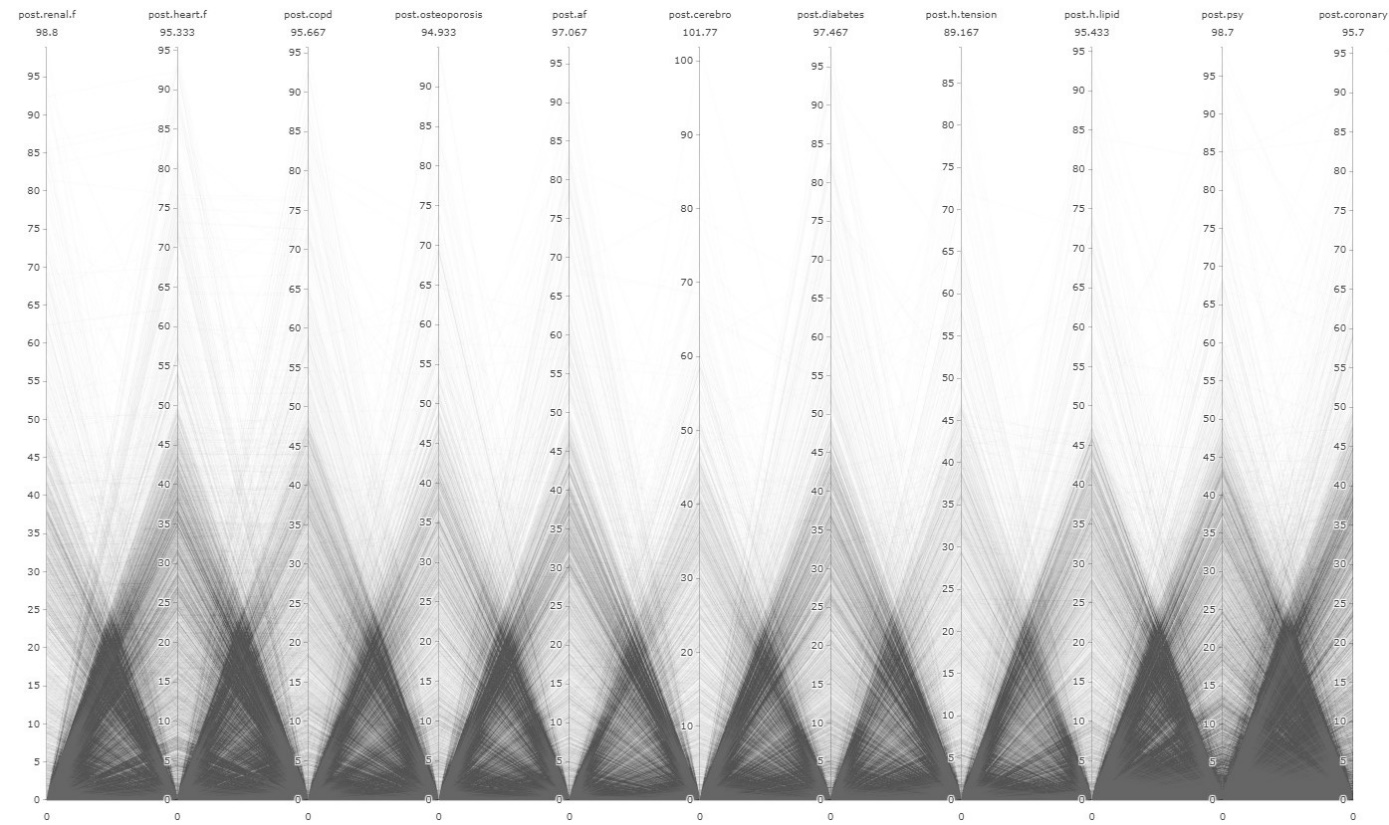


**Annex 12: Relational Analysis of Diagnosed Diseases of Individuals Diagnosed with Dementia in the Postclinical Process (In Regions Have Higher Dementia Rates than National Average)**
