# Supplementary material for: Insights from Turkey's big data: unraveling the preventability, pathogenesis, and risk management of Alzheimer's disease (AD)
Source: Sci Rep. 2024 Mar 12;14:6005. doi: 10.1038/s41598-024-56702-1 (PMC10933367; doi:10.1038/s41598-024-56702-1)
Supplement: Supplementary file 6 — Supplementary Information 6. [file 41598_2024_56702_MOESM6_ESM.docx]

**This file includes R Studio codes for the PCG analysis in the study.**

install.packages("plotly")

library(plotly)

df <- pcg

fig <- df %>%

plot_ly(width = 1500, height = 900)

fig <- fig %>% add_trace(type = 'parcoords',

line = list(

colorscale = 'viridis',

showscale = TRUE,

reversescale = TRUE,

cmin = -4000,

cmax = -100),

dimensions = list(

list(range = c(~min(post.renal.f),~max(post.renal.f)),

constraintrange = c(100000,150000),

label = 'post.renal.f', values = ~post.renal.f),

list(range = c(~min(post.heart.f),~max(post.heart.f)),

label = 'post.heart.f', values = ~post.heart.f),

list(range = c(~min(post.copd),~max(post.copd)),

visible = TRUE,

label = 'post.copd', values = ~post.copd),

list(range = c(~min(post.osteoporosis),~max(post.osteoporosis)),

label = 'post.osteoporosis', values = ~post.osteoporosis),

list(range = c(~min(post.af),~max(post.af)),

label = 'post.af', values = ~post.af),

list(range = c(~min(post.cerebro),~max(post.cerebro)),

label = 'post.cerebro', values = ~post.cerebro),

list(range = c(~min(post.diabetes),~max(post.diabetes)),

label = 'post.diabetes', values = ~post.diabetes),

list(range = c(~min(post.h.tension),~max(post.h.tension)),

label = 'post.h.tension', values = ~post.h.tension),

list(range = c(~min(post.h.lipid),~max(post.h.lipid)),

label = 'post.h.lipid', values = ~post.h.lipid),

list(range = c(~min(post.psy),~max(post.psy)),

label = 'post.psy', values = ~post.psy),

list(range = c(~min(post.coronary),~max(post.coronary)),

label = 'post.coronary', values = ~post.coronary)

)

)

fig
